# Personalized Targeted Therapy for Lung Cancer

**DOI:** 10.3390/ijms130911471

**Published:** 2012-09-13

**Authors:** Kehua Wu, Larry House, Wanqing Liu, William C.S. Cho

**Affiliations:** 1 Department of Medicine, University of Chicago, Chicago, IL 60637, USA; E-Mails: kwu@bsd.uchicago.edu (K.W.); lhouse@medicine.bsd.uchicago.edu (L.H.); 2 Department of Medicinal Chemistry and Molecular Pharmacology, College of Pharmacy, Purdue University, West Lafayette, IN 47907, USA; E-Mail: liu781@purdue.edu; 3 Department of Clinical Oncology, Queen Elizabeth Hospital, Hong Kong, China

**Keywords:** ALK, biomarker, EGFR, lung cancer, next-generation sequencing

## Abstract

Lung cancer has long been recognized as an extremely heterogeneous disease, since its development is unique in every patient in terms of clinical characterizations, prognosis, response and tolerance to treatment. Personalized medicine refers to the use of markers to predict which patient will most likely benefit from a treatment. In lung cancer, the well-developed epidermal growth factor receptor (EGFR) and the newly emerging EML4-anaplastic lymphoma kinase (ALK) are important therapeutic targets. This review covers the basic mechanism of EGFR and EML4-ALK activation, the predictive biomarkers, the mechanism of resistance, and the current targeted tyrosine kinase inhibitors. The efficacy of EGFR and ALK targeted therapies will be discussed in this review by summarizing the prospective clinical trials, which were performed in biomarker-based selected patients. In addition, the revolutionary sequencing and systems strategies will also be included in this review since these technologies will provide a comprehensive understanding in the molecular characterization of cancer, allow better stratification of patients for the most appropriate targeted therapies, eventually resulting in a more promising personalized treatment. The relatively low incidence of EGFR and ALK in non-Asian patients and the lack of response in mutant patients limit the application of the therapies targeting EGFR or ALK. Nevertheless, it is foreseeable that the sequencing and systems strategies may offer a solution for those patients.

## 1. Introduction

Lung cancer has the highest mortality rate of all cancers, and is the second most diagnosed cancer in both men and women, behind prostate and breast cancer, respectively [1–3]. It has been reported that more than 1.6 million cases are diagnosed each year along with 1.3 million deaths [3]. Recently, the United States has released cancer statistics showing an occurrence of 221,130 new cases of lung cancer in 2011, accounting for ~14% of all cancer cases expected to be diagnosed. Approximately 85%–90% of lung cancer cases are caused by voluntary or involuntary (second hand) cigarette smoking [4]. Lung cancer is mainly divided into two classes, which are non-small cell lung cancer (NSCLC, ~85%) and small cell lung cancer (SCLC, ~15%), according to biology therapy and prognosis [1]. NSCLC could be further divided into squamous cell carcinoma (SCC), adenocarcinoma and large cell lung carcinoma (LCLC). Genetic susceptibility plays a crucial role in the occurrence of lung cancer, especially in young patients [2].

Lung cancer is aggressive and its treatment remains one of the most challenging tasks in the medical world. Conventional treatment modalities include surgery, radiation therapy and chemotherapy. The selection of therapy is dependent upon the cancer type (small cell or non-small cell), development stage, and genetic characterization. Patients diagnosed with lung cancer often receive more than one type of treatment. The discovery and development of molecular inhibitors have had a major impact in the treatment of NSCLC [5]. In the last decade, four molecular targeted agents were approved for treatment of lung cancer: gefitinib (2002), erlotinib (2003), bevacizumab (2006), and crizotinib (2011) [6]. The 1-year survival rate for lung cancer was 43% in 2003–2006. However, the overall 5-year survival rate for all stages is still as low as 16%–17% for NSCLC and even lower in SCLC (6%). Despite the 5-year survival rate reaching 53% for patients diagnosed at an early stage, only 15% of such cases were determined in a timely manner when the tumor was still localized [2].

Cancer has long been recognized as an extremely heterogeneous disease, since its development is unique in every patient in terms of clinical characterizations, prognosis, response and tolerance to treatment [7,8]. The inception of the human genome project gave birth to “personalized medicine” in cancer care treatment. This revolutionary method promises to help patients improve outcomes, avoid unnecessary treatments and reduce health care cost [8]. In oncology, the term “personalized medicine” refers to moving away from a “one size fits all” strategy in cancer therapy by the use of identifying markers that reliably predict which patient will most likely benefit from treatment [9]. Over the past 20 years, research in targeted treatment has focused on novel treatment programs. Personalized targeted therapy has already been applied towards the treatment of various cancers such as: NSCLC, squamous-cell carcinoma of the head and neck [10], colorectal cancer [11], pancreatic cancer [12], and breast cancer [13]. In the future, cancer care targeted at genes and proteins of tumor cells may assist in early detection and help physicians design the most appropriate treatment package tailored to each individual patient.

In this review, we will summarize the pathways, mechanisms, and the current corresponding inhibitors for epidermal growth factor receptor (EGFR), as well as the newly emerging EML4-anaplastic lymphoma kinase (ALK) targets. The efficacy of tyrosine kinase inhibitors (TKIs) were evaluated by summarizing prospective validating biomarker-based clinical trials. Revolutionary sequencing and systems strategies will also be reviewed since we expect these methodologies to make personalized medicine a reality by looking at whole genome sequencing and the possible aberrations, rather than just one target (such as EGFR, KRAS or EML4-ALK).

## 2. Molecular Targets

### 2.1. EGFR

**Figure 1:**
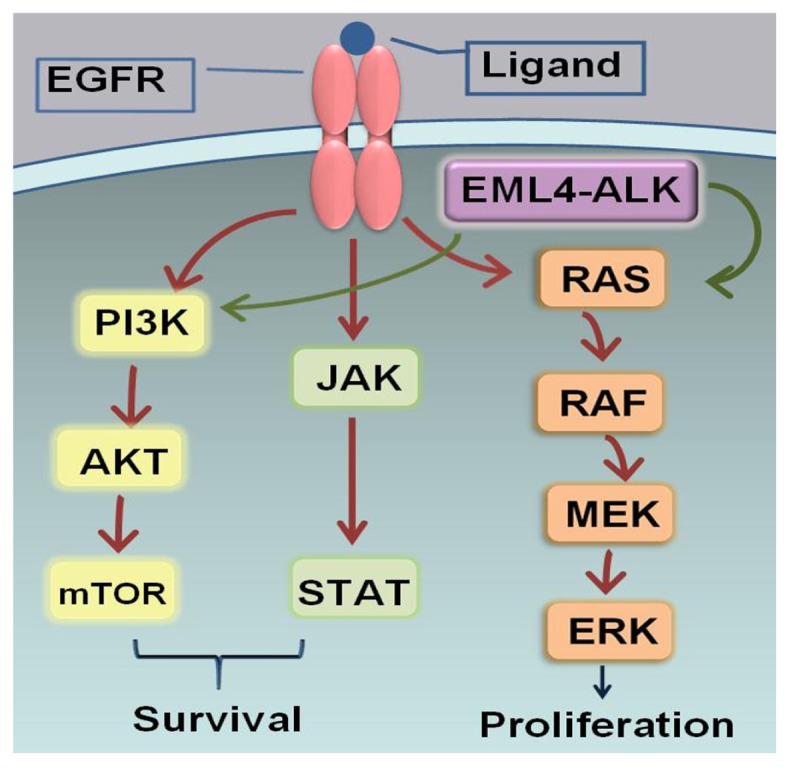
Epidermal growth factor receptor (EGFR). The binding between EGFR and ligand triggers downstream intracellular signaling pathways including the PI3K/AKT prosurvival, STAT transcription, and RAS/RAF/MEK proliferation pathways. The anaplastic lymphoma kinase (ALK) fusion proteins mainly activate the RAS/RAF/MEK and PI3K/AKT pathways. Amplification of the EGFR and ALK signaling pathways drives cell proliferation, cell motility, and carcinogenesis.

EGFR is a member of the ErbB family of cell surface receptor tyrosine kinase (RTK). The EGFR family consists of four members: EGFR (or ErbB-1), HER-2 (or ErbB-2), HER-3 (or ErbB-3), and HER-4 (or ErbB4) [14]. It has been demonstrated that RTKs play a crucial role in tumorigenesis by controlling signal transduction pathways that regulate proliferation and apoptosis [15]. The RTKs (with the exception of HER-2) are activated by the binding of specific activating soluble ligands, which occur in the extracellular portion of the RTKs [16]. This interaction between ligands and receptors promotes the formation of functional active homodimers (EGFR dimer) or heterodimers (HER3 or HER4 dimer). It also activates the intracellular kinase domain and a subsequent ATP-dependent cross-autophosphorylation of *C*-terminal tail of the receptor [14,17]. Eventually, the phosphorylated residues recruit a diverse set of cytoplasmic signaling molecules as a docking site, which triggers downstream intracellular signaling pathways including the PI3K/AKT prosurvival, STAT transcription, and RAS/RAF/MEK proliferation pathways (Figure 1) [18,19]. It is virtually sufficient to deregulate the signaling pathways by stimulating apoptosis and growth cessation [19,20]. In NSCLC patients, 50%–80% have shown an over-expression of EGFR [21], which is associated with angiogenesis and poor prognosis [22]. The association between EGFR alteration and pathogenesis makes it a prime candidate for targeted treatments.

**Figure 2:**
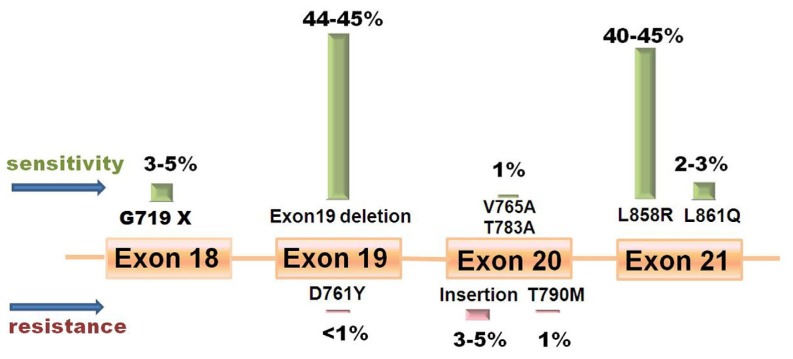
The frequency of EGFR mutations. The deletion of exon 19 nested located between residues 747–750, which are mainly composed of delGlu746-Ala750, delGlu746-Ser752insVal, delLeu747-Thr751, delLeu747-Ser752, and delLeu747-Pro753insSer.

Potent response predictors are necessary to help doctors predict which patients will most likely to respond to EGFR TKIs. These predictors are ultimately used to obtain an optimal treatment while avoiding resistance. In 2004, three influential studies discovered a series of somatic mutations in the kinase domain of EGFR which are associated with the response to EGFR TKI therapy [23–25]. To date, EGFR mutations are presumed to be the strongest predictive biomarker for the efficacy of EGFR TKI therapy [26] due to much higher response rate (RR, 37.5%–100% *vs.* 2.9%–23% [27]; 70% *vs.* 33.2% as a first-line treatment; 47.4% *vs.* 28.5% as a second-line treatment [28]) and longer overall survival (OS, 13–23 months *vs.* 5–17 months [27]) in mutant patients. Mok [29] summarized six clinical trials to compare the response to EGFR TKIs and chemotherapy in patients carrying positive mutations. Patients have responded better to EGFR TKIs than to chemotherapy demonstrated by a higher RR (62.1%–84.6% *vs.* 10.5%–47.3%) and longer progression-free survival (PFS) (8.4–13.1 months *vs.* 4.6–6.7 months). In April 2011, the American Society of Clinical Oncology (ASCO) has issued a provisional clinical opinion, which suggested that initiating first-line therapy with an EGFR TKI should be based on positive EGFR mutation tests in patients with newly diagnosed advanced NSCLC [30]. EGFR mutations are more common in non-smoking East Asian females and those with adenocarcinoma histology (95% were found in adenocarcinomas) [31–36]. There are several reviews summarizing the frequency and distribution of EGFR mutations (Figure 2) [14,15,29,33,37–39].

EGFR gene copy number is also considered to be a good predictor for response to EGFR TKI therapy. It has been demonstrated in several studies that an increased copy number is associated with a higher overall RR, a longer PFS, and an OS benefit during treatment with erlotinib or gefitinib [40–42]. In fact, EGFR mutation was validated to be more selective than EGFR gene number [43].

### 2.2. EML4-ALK

The ALK tyrosine kinase receptor has gained much attention recently as a newly emerging relevant biomarker and therapeutic target in NSCLC. ALK is one of the members of the insulin receptor family located at chromosome 2 and encodes a trans-membrane receptor tyrosine kinase [44,45]. The activation of ALK is primarily through the formation of fusion genes (Figure 1) [46]. EML4-ALK translocation is the most common ALK gene rearrangement [47]. The intracellular kinase domain of ALK fuses with the *N*-terminal of EML4, and then encodes a cytoplasmic chimeric protein with kinase activity, which subsequently drives tumor growth [47]. EML4-ALK rearrangements in NSCLC patients are mainly found in younger non-smoking patients with adenocarcinoma [48,49]. EML4-ALK rearrangements are mutually exclusive with EGFR or KRAS mutations [47,50]. It has been reported that approximately 2%–11% of tumors carrying positive EML4-ALK, which is rarely found in SCC [33,36,51,52].

### 2.3. KRAS

KRAS mutations are a negative predictor of response to EGFR TKIs, mainly accounting for primary resistance [53]. Most KRAS mutations in lung adenocarcinoma are associated with smoking. KRAS positive mutations are limited to NSCLC (predominantly adenocarcinomas) and are mutually exclusive to mutations in EGFR and ALK [54]. In several countries, patients harboring a KRAS mutation have been excluded from EGFR TKI therapy [55].

### 2.4. The Potential Targets under Development

Mammalian target of rapamycin (mTOR), with serine/threonine kinase activity, appears to trigger the activation of PI3K pathway through ligand binding and eventually regulates the cell cycle. The development of mTOR inhibitors provides a great opportunity for helping patients with solid tumors. To date, studies utilizing mTOR inhibitors in NSCLC patients have reached phase I/II clinical trials as a monotherapy or in combination. These mTOR inhibitors are known as sirolimus, temsirolimus, everolimus, and others [56].

The amplification of fibroblast growth factor receptor 1 (FGFR1), predominantly in SCC (up to ~20%), is considered to be a potential target for treatment with anti-FGFR1 agents [57]. Dy *et al*. reported dose-dependent tumor cell death (6 out of 9 lung cancer cell lines) due to the treatment of Y15 (1,2,4,5-benzentetraamine tetra hydrochloride). Y15 is a small molecular inhibitor of focal adhesion kinase (FAK), which is a non-receptor tyrosine kinase [58]. Mutations in the DDR2 kinase gene were indicated to drive SCC. The cell lines harboring DDR2 mutations were sensitive to dasatinib, which blocked cellular transformation. DDR2 mutations are found in 4% of SCC patients [59].

## 3. Resistance

Some of the patients who have benefited from EGFR TKI therapy eventually generated resistance to the drug. This acquired resistance to TKI therapy is mostly due to a specifically acquired EGFR mutation known as T790M (at exon 20) [60]. T790M mutations account for more than 50% of acquired resistance in adenocarcinomas [33]. Besides the T790M mutation, the amplification of MET also activates similar downstream pathways, which accounts for 20% of acquired resistance [61].

The underlying mechanism for the remaining 30%–40% of resistance to EGFR TKIs is unknown. Ogawa *et al*. [62] found that the death-associated protein kinase (DAPK) is hypermethylated in resistant cells. The generation of a population of cancer cells with stem cell properties might be another possible reason of resistance to EGFR TKIs [63].

Patients treated with ALK inhibitors may acquire a resistance similar to patients taking an EGFR TKI. Choi *et al*. [64] have found secondary mutations within the kinase domain of EML4-ALK in tumor cells along with acquired resistance. The tumor cells harboring either C1156Y or L1196M mutations had a very low response to ALK inhibitors. L1196M mutant cells were more resistant to crizotinib than C1156Y mutant cells. The mutations in the *PIK3CA* gene and histologic change from NSCLC to SCLC were also found to be potential resistance mechanisms [65].

## 4. Targeted Agents

The main approach to block the EGFR pathway is by competing with ATP for binding to the tyrosine kinase domain. The EGFR TKIs are summarized in Table 1. Gefitinib and erlotinib are reversible inhibitors of the EGFR kinase and are also called “first-generation” small molecular inhibitors. Gefitinib was the first targeted agent entered into clinical trials currently approved by the FDA. Gefitinib “should be used only in cancer patients who have already taken the medicine and whose doctor believes it is helping them” [66]. New patients should not be given this drug due to a lack of OS benefit as shown in the ISEL trial [67]. Gefitinib is now widely prescribed in Asia. Erlotinib has received global approval as the treatment in second-line and third-line therapy. The first-generation of reversible EGFR TKIs usually generated resistance within one-year of treatment [68] prompting the development of a second-generation (Table 1). The second-generation TKIs may overcome resistance to the treatment of erlotinib or gefitinib via the T790M gatekeeper mutation. However, this activity needs to be further validated since it has also been reported that afatinib, a second-generation TKI, was not qualitatively superior in preventing the acquired resistance [69]. Several irreversible EGFR inhibitors blocked multiple EGFR family members, interrupting the cooperative signal pathway among EGFR members and resulted in a more complete blockage. It is not surprising that dacomitinib (PF299804) has a significantly longer PFS than erlotinib (*p* = 0.017) in patients carrying the wild type EGFR, since it‘s a potent irreversible inhibitor of EGFR, HER2, and HER4 [70]. The second-generation EGFR TKIs may have better efficacy as well as a delayed resistance, and may work in patients resistant to reversible inhibitors. There are also multiple pathways inhibitors at various clinical stages, which are shown in Table 1.

Crizotinib had received accelerated approval by the FDA, in August 2011 to treat patients diagnosed with late-stage (locally advanced or metastatic) NSCLC carrying positive ALK rearrangement. The corresponding diagnostic test method, Vysis ALK Break Apart FISH Probe Kit, was approved in tandem. AP26113 is a potent dual small-molecule inhibitor of ALK and EGFR (including T790M). A phase I dose escalation trial was initiated on Sep 2011 (NCT01449461), and phase II clinical trials with selected patients are expected to start in late 2012 according to the ARIAD website [71]. LDK378 is a selective small molecule ALK inhibitor. Preliminary responses were observed in its first-in-human trial [72]. Additional ALK inhibitors are summarized in Table 2.

## 5. Personalized Clinical Practices in Biomarker-Based Selected Patients

The last few decades have seen remarkable progress in target biomarker discovery, validation, biomarker measurement, research and development of targeted agents, and preclinical/clinical studies evaluating the efficacy of target inhibitors. Eventually, the prospective clinical trials in selected patients stratified by a target will bring personalized treatment from promise to reality.

### 5.1. Selecting Patients Based on Histology

The response to EGFR TKIs was initially thought to be related to the clinical characterization of patients, such as Asian female non-smokers with adenocarcinomas. There were studies focused on the evaluation of efficacy in these patients. Rizvi *et al*. [73] completed a clinical trial in patients who were enriched with the EGFR mutation (less than 15 packs per year for their cigarette smoking history and/or a component of bronchioloalveolar carcinoma (BAC)). The overall RR in all eligible patients was 42% (21 out of 50), while the RR in the mutation patients was 81% (17 out of 21). A phase II trial of erlotinib was completed recently. Forty-nine patients, who had stage III B/IV pulmonary adenocarcinoma or BAC and were non-smokers or former light smokers, were enrolled in this study. The overall RR was 25.5% in all patients, 66.7% in mutant patients, and 14.8% in wild type patients. Milella *et al*. reported a prospective phase II study in which patients were divided into four groups (EGFR mutation; highly polysomic/amplified EGFR; EGFR and/or pAKT positive; adenocarcinoma/BAC with a non-smoking history) and were given EGFR TKIs as second or subsequent line treatment. The 1st and 4th groups attained the best and second best overall RR (25% and 20%, respectively), disease control was highest in group 1 and group 4 (>50%), PFS and OS (*p* = 0.02 and 0.01, respectively) [74]. The selection of patients should be based on EGFR mutation. Clinical characterization can be a secondary criterion if EGFR mutation is not available.

### 5.2. Targeted Treatment in EGFR Mutant Patients

EGFR is the most developed target in lung cancer. Many preclinical and clinical studies have demonstrated that EGFR mutations are potent and selective prediction biomarkers for treating patients with EGFR TKIs. The discovery and validation of targets, including EGFR mutations, enables us move toward the eventual goal of personalized treatment based on the uniqueness of each patient. We have summarized the clinical trials in biomarker-based selection of patients.

The Biomarker-integrated Approaches of Targeted Therapy for Lung Cancer Elimination (BATTLE) trial in NSCLC was a pioneering phase II clinical trial to demonstrate the use of biomarkers to guide the treatment of patients with advanced NSCLC refractory given prior chemotherapy [75]. The BATTLE trial was more focused on the patient and utilized real-time biopsies revealing the uniqueness of each tumor and provided physicians a powerful tool for giving targeted therapies which are most likely to show efficacy. A total of 244 patients were eligible for this study and the first 97 patients were equally randomly assigned into 4 groups which were given erlotinib, vandetanib, erlotinib plus bexarotene and sorafenib, respectively. Eleven potential biomarkers were identified in the eligible patients. Once a satisfactory number of baseline results had been collected, the remaining 158 patients were assigned to treatment arms which were most likely going to give the best response based on their tumor type. The overall 8-week disease control rate (DCR) was 46% (34% for erlotinib; 33% for vandetanib; 50% for erlotinib/bexarotene and 58% for sorafenib); median PFS was 1.9 months; median OS was 35%. The 8-week landmark analysis indicated that median survival with 8-week disease control was 9.6 months, while it is 7.5 months without 8-week disease control. The major findings from the BATTLE trial reported that patients given treatment based on their tumor biomarkers showed more benefit compared to patients with unselected therapy. An effective treatment-marker-group pairing (defined as 0.8 posterior probability of exceeding a DCR of 30%) revealed that combinations of erlotinib in the VEGF/VEGFR-2 group; vandetanib in the EGFR group; erlotinib/bexarotene in EGFR, retinoid X receptor, cyclin D1, and non-marker groups; sorafenib in KRAS/BRAF, VEGF/VEGFR-2 and no marker group. An analysis of individual markers for treatment efficacy has demonstrated that EGFR mutation rates can (1) predict the response to erlotinib (*p* = 0.04), (2) predict a high VEGFR-2 expression for vandetanib (*p* = 0.05), and (3) predict a high cyclin D1 expression for erlotinib/bexarotene (*p* = 0.01). These predictions confirmed the authors’ hypotheses that biomarkers could better predict 8-week disease control. “The BATTLE trial represents an important model for future genotype-driven studies of targeted therapies in lung cancer,” says William Pao. Integrating the results of real-time biomarker detection, the BATTLE trial is moving towards tailoring treatment in specific patient populations for desirable individualized treatment. “Ultimately, we would like to be able to screen patients for tumor characteristics and give them appropriate therapies up front” (Dr. Edward S Kim). The following BATTLE-2 trial is ongoing.

We summarized recent clinical trials with selected patients that carry positive EGFR mutations in Table 3. The efficacy and toxicity of EGFR TKIs as first-line treatment was compared with chemotherapy in several phase III clinical trials (Table 3). Prospective trials comparing the efficacy of chemotherapy and EGFR TKIs in EGFR-mutant patients can provide further insight on the most appropriate treatment in this population. NSCLC Patients from Europe [76], China [77], and Japan [78] carrying positive EGFR mutations were enrolled in the studies. Erlotinib or gefitinib was compared to cisplatin/docetaxel or gemcitabine/carboplatin. Two [76,78] of the three studies found significant longer PFS (9.7 *vs.* 5.2 months, HR 0.37, *p* < 0.0001; 9.2 months *vs.* 6.3 months, HR 0.489, *p* < 0.0001) and higher RR (64% *vs.* 18%) in EGFR TKIs treatment group (Table 3). The another study [77] compared the two groups with PFS, which is longer in the erlotinib group compared to the chemotherapy group (13.1 *vs.* 4.6 months, HR 0.16, *p* < 0.0001). Chemotherapy resulted in more grade 3 or 4 toxicity compared to erlotinib [76–78]. The efficacy of EGFR TKIs’ therapy is consistent among the three populations, indicating the absence of ethnic differences. The EURTAC trial was among the first prospective head-to-head phase III study along with the other two studies. It has validated the ASCO proposal considering routine pretreatment assessment of EGFR mutations in patients with NSCLC [76]. LUX-lung 3, the largest prospective trial in EGFR mutation positive lung cancer using pemetrexed/cisplatin as a comparison, indicating that afatinib might be a potent first-line treatment in EGFR positive patients due to the improved PFS (11.1 months *vs.* 6.9 months) [79]. A better response can be expected when EGFR mutant patients are treated with EGFR TKIs rather than chemotherapy. The OS is usually not available in two arm controlled studies due to a common crossover or carryover effect [29,80].

The efficacy and safety of EGFR TKIs as a first-line treatment were prospectively evaluated in biomarker-based selected patients (Table 3). The RR ranged from 53.3% (Korean patients) [81] to 75% (Japanese patients) [82]. The PFS was 7.1 months to 398 days. The incidence of grade 3 toxicity is ~13% [82]. DCR was from 86.7% [81] to 96% [82]. The OS was also observed in two of these studies (819 days, 17.5 months) [81,83], approximately two-fold higher than chemotherapy in unselected NSCLC patients [83]. Median survival time is 17.8 and 20 months [84,85]. Moreover, Han *et al*. [86] demonstrated that erlotinib as neoadjuvant treatment in patients with stage IIIA-N2 NSCLC and with an activating EGFR mutation is feasible. Inoue *et al*. [84] observed the response to gefitinib treatment in EGFR mutation-positive patients with extremely poor performance status. This was the first reported observation showing patients receiving benefit from gefitinib in this population. This favorable response further validates biomarker-based treatment selection is feasible and proves that EGFR mutant patients can benefit from this selection strategy. The PFS of erlotinib and gefitinib monotherapy in EGFR mutant patients (Table 3) is comparable (9.7–13.1 months *vs.* 7.1–13.3 months). The RR ranged from 53.3% to 75% and the DCR ranged from 86.7% to 96% [81–88].

There are two biomarker-guided clinical studies, in which patients were assigned to either an EGFR TKI group or a chemotherapy group based on their EGFR mutation status (Table 3) [89,90]. Patients were given erlotinib or gefitinib if they were carriers of the EGFR mutation, while those with wild type EGFR received chemotherapy with/without cisplatin (depending on the BRCA1 mRNA levels). EGFR mutant patients responded to erlotinib/gefitinib better than patients with the wild type EGFR.

Pietanza *et al.* [91] and Sequist *et al.* [92] reported on the efficacy of XL647 and neratinib, respectively, in patients who either had resistance or generated resistance from prior treatment, but neither of these studies reported positive results (Table 3). There are also many ongoing biomarkerbased clinical trials, such as the PROSE trial [93] and UMIN 000005086 [94].

### 5.3. Clinical Trials in Patients with Wild Type EGFR

A limited EGFR mutation incidence prompted researchers to conduct studies to evaluate the efficacy of EGFR TKIs in patients with wild type EGFR (Table 4). Kobayashi *et al*. [95] concluded that “EGFR-TKI using erlotinib may be an alternative option for patients resistant to cytotoxic chemotherapy, even in those with EGFR wild-type NSCLC” based on the results from a phase II clinical trial in patients carrying wild type EGFR (Table 4). A similar result could be found in another clinical trial, which was performed by Matsuura *et al*. [96] in 2011. Erlotinib was given to patients carrying wild type EGFR as a third-line treatment. The acceptable RR (15%) and OS (6.7 months) indicated that erlotinib could be a potential third-line treatment option for patients without an EGFR mutation. Yoshioka *et al*. [97] also reported a phase II clinical trial in which Japanese patients carrying the wild type EGFR previously received one to three chemotherapy regimens. The objective RR was lower than the authors had initially expected. The efficacy of erlotinib in wild type EGFR patients is limited to this study. Garassino *et al*. [98] compared erlotinib with docetaxel and found that docetaxel is superior to erlotinib as a second line treatment. The treatment of EGFR TKIs in patients with EGFR heterogeneity was controversial according to the above prospective clinical studies, which is consistent with the conclusions from previous retrospective studies. The IPASS study has demonstrated that non-mutant patients receiving gefitinib have inferior outcomes (HR 2.85, *p* < 0.001) [100]. On the other hand, the BR.21 trial [101] discovered that patients carrying the wild type mutation seemed to benefit from the administration of erlotinib compared to the placebo arm (not significant). Sharma *et al*. [15] found that approximately 10%–20% of lung cancer patients without activating EGFR mutations had a partial response to gefitinib. Overall, the usage of EGFR TKIs in patients without a mutation needs to be studied more to confirm the efficacy or inefficacy.

Metro *et al*. [99] finished a clinical trial in the patients carrying wild type EGFR. The relationship between KRAS mutation status and the response to EGFR-TKI was observed in this trial. The median PFS was 1.6 months in KRAS mutants group, while it was 3.0 months in KRAS wild type group, indicating wild type EGFR plus mutant KRAS were associated with an increased resistance. Moreover, the patients with KRAS codon 13 mutants experienced worse responses than the patients harboring KRAS codon 12 mutants, which revealed that specific KRAS oncogene substitutions may lead to differential sensitivity and should be considered when predict the resistance/sensitivity.

### 5.4. Selecting Patients Based on KRAS Mutation

Janne *et al*. [102] presented their clinical study on KRAS positive patients at the 2012 ASCO meeting. It was the first prospective study to evaluate the clinical benefit of a targeted therapy for patients with KRAS mutant cancer. There were 422 patients enrolled in this study with stage IIIB-IV KRAS mutant NSCLC, who had received prior chemotherapy. Two treatment combinations compared selumetinib (AZD6244, ARRY-142866) + docetaxel *vs.* docetaxel + placebo. OS was longer in SEL/DOC group (9.4 months *vs.* 5.2 months; without significant difference). The patients in SEL/DOC group had significant better RR (DOC 0%, SEL/DOC 37%, *p* < 0.0001) and PFS (DOC 2.1 months, SEL/DOC 5.3 months, *p* = 0.0138) than those were given DOC alone, indicating that a KRAS mutant patient can benefit from targeted therapy (SEL + DOC). The BATTLE trial demonstrated that KRAS mutation tumors benefited from the therapy of sorafenib [75]. Riely *et al*. found that ridaforolimus, an inhibitor of mTOR, was associated with prolonged PFS in KRAS mutant patients compare with placebo (4 months *vs.* 2 months, *p* = 0.013, HR 0.36). [103]

### 5.5. Selecting Patients Based on ALK Rearrangement

The development of ALK inhibitors has been more rapid than EGFR TKIs. There are two reports considering the PROFILE 1005 trial during the ASCO meeting for two consecutive years [104,105]. The ongoing latter global clinical study in NSCLC patients with ALK-rearranged indicates that crizotinib gives a high RR, good PFS, modest toxicity, and improvement in patient-reported symptoms [105]. A phase I trial performed by Shaw *et al*. [106] was also a biomarker guided study. Patients with positive ALK were divided into a crizotinib treatment group and a control group. In the latter group, patients were treated with any second-line therapy. The authors show that crizotinib-treated ALK-positive patients had a higher OS for 1–2 years compared to the ALK-positive control group. The survival in crizotinib-treated ALK-positive patients was similar to the EGFR mutant group who were treated by EGFR TKIs indicating that ALK positive patients like EGFR mutant patients would benefit from a strategy looking at these biomarkers.

## 6. Sequencing and Systems Strategies

After the completion of the Human Genome Project in 2003, great improvement in genome-wide mapping technologies, such as next-generation sequencing (NGS), lead to decreased time and financial cost. This technological improvement makes it affordable and practical, leading to ‘sequencing and systems strategy’ [107]. It eases clinical decision making to match patients with the appropriate targeted treatment based on their genome information. The specific pathway targeted therapy as described above, relies on one (or a few) favorite genes. The sequencing and systems strategy is treating cancer differently by sequencing the whole genome using both tumor and normal tissue, surveying the global landscape of cancer, and then matching therapies to targets based on all the possible aberration [107]. Whole-genome sequencing (WGS) of primary tumors and matched metastatic biopsies provides a novel systematic discovery of mutational spectra underlying tumors, resulting in very rich genomic data. This informative genome data set could offer a more systematic consideration of treatment since cancer is usually associated with a variety of genetic alterations (such as structural abnormality, copy number gain, somatic single nucleotide variants, *etc*.). The thorough understanding of this diverse heterogeneity in the cancer genome sequence may potentially help to build a mutation-based taxonomy, shape personalized therapy [107], and predict the risk of developing a cancer [108].

Several WGS studies have been completed in lung cancer. Over 30 sites of significant somatic copy number alteration were identified in the Cancer Genome Atlas, a large-scale collaborative study, among 178 patients with SCC histology. Exome sequencing revealed 13 significantly mutated genes (false discovery rate < 0.01). The high expression was found in TP53, CDKN2A, PTEN, KEAP1, and NFE2L2. Four distinct expression subtypes, NFE2L2 and KEAP1 mutations, FGFR kinase alterations, increased global methylation and the highest rate of tobacco use, were identified by mRNA expression profiling. Whole genome shotgun sequencing detected the rearrangements involving several known tumor suppressors in 20 tumor/normal sample pairs, which were further confirmed by RNA sequencing including PTEN, RB1, NOTCH1, NF1, and CDKN2A. It was found that 75% patients (127 out of 178) had potential therapeutic targets [109]. Lee *et al*. [110] performed the NGS of a primary lung tumor and adjacent normal specimens from a 51-year-old male Caucasian patient, who had NSCLC and smoked for 15 years. More than 50,000 single nucleotide variants were identified in non-expressed genes and promoter regions, accounting for 17.7% of WGS data. The authors validated 530 variants, in which one lied in KRAS proto-oncogene, other 391 variants were found in coding regions, and 43 variations were structural alterations. Ju *et al*. [111] reported an integrated analysis of massively parallel whole-genome and transcriptome sequencing for a paired cancer/normal tissue from a young non-smoking patient with adenocarcinoma. This study discovered that the fusion of KIF5B and RET might result in a subset of NSCLC, indicating that a chimeric oncogene would make a potentially promising molecular target for diagnosis and personalized treatment. A WGS approach could also explore the prognostic indicator. Belvedera *et al*. [112] generated a computational index (GH index), which is derived from whole-genome copy number analysis, to evaluate the overall genomic damage. GH index showed a potential value to predict prognostic in SCC patients and may stratify patients better.

Genome-wide mapping provides a good opportunity to design clinical trials more thoughtfully and tailor individual treatments more evidently. While it also presents several logistical challenges, including the selection of exome or whole-genome sequencing; distinction between real drivers and disturbing passengers; bioinformatic support for the interpretation of genomic data and the eventual clinical implementation [113]. Currently, the studies utilizing this technology are usually performed as international collaborative for many common cancers, due to the time and cost-effective concerns [108].

## 7. Perspective

Overall, the landscape in lung cancer is rapidly developing with the discovery and identification of new molecular targets as well as new therapies that specifically inhibit cancerous activity. The integrated pipeline from discovery of a target to the biomarker-based treatment selection brings personalized medicine into reality. There is abundant evidence showing that patients who carry these targets can benefit from the treatment of corresponding inhibitors such as EGFR mutation and ALK arrangements. In this review, we summarized the currently completed clinical practices based on selected patients. These clinical trials have shown promising RR, ideal PFS and modest adverse effects, indicating that the biomarker-directed selection strategy is feasible in patients with a positive mutation. Most of the clinical trials had investigated erlotinib and gefitinib. The second-generation EGFR TKIs and the newly emerging ALK inhibitors have not been fully validated yet.

On the other hand, the limitation of EGFR and EML4-ALK inhibitors should not be ignored. The treatment of EGFR TKIs in patients harboring positive mutations led to a RR of 50%–75%. The intrinsic reason for a lack of response in the target population is unknown, which may improve the efficacy of targeted therapy greatly. EGFR and EML4-ALK mutations attribute to ~28% and ~11% [36] in American non-smokers and the incidence is even lower in smokers. Patients without these mutations (~87%) will not benefit from these particular inhibitors. This phenomenon has limited the overall progress in lung cancer therapy. Moreover, the activating targets are mainly found in adenocarcinoma, a NSCLC. The targeted therapy for SCC, LCLC and SCLC will need to be developed to make a broader impact on NSCLC. The whole-genome technology and the corresponding “sequencing and systems strategy” may be the key to this situation. This technology will very likely provide us with a comprehensive understanding in molecular characterization of cancer, allow us stratify patients more intelligently and match targeted therapy to the right patients, eventually resulting in a fulfilled and fully promising personalized treatment.

Since there are numerous ongoing research and clinical trials, we will expect more potent targets and biomarkers to be discovered and validated giving optimism that more targeted agents will be developed in the near future.

## Figures and Tables

**Table 1:** Summary of EGFR TKIs for NSCLC.

Agent	Molecular properties	Approved status	Company
**First-generation**			

Iressa/gefitinib	EGFR	Marketed in over 64 countries. It is a third-line treatment of NSCLC, after platinum-and docetaxel-based chemotherapy failed.	AstraZeneca
Tarceva/erlotinib	EGFR	Approved by several agencies, including U.S. Food and Drug Administration and European Medicines Agency, as second- and third-line treatment of NSCLC after platinum-based chemotherapy failed.	OSI/Roche/Genentech
Icotinib	EGFR	State Food and Drug Administration of China approved for the treatment of patients with advanced stage NSCLC.	Zhejiang Beta Pharma

**Second-generation**			

Afatinib/BIBW 2992	EGFR/T790M	Phase III clinical trial	Boehringer-Ingelheim
Dacomitinib/PF299804	Pan-EGFR/T790M	Phase III clinical trial	Pfizer
Neratinib/HKI-272	EGFR, HER1, HER2	Phase I/II clinical trial	Pfizer
AP26113	EGFR/T790M, ALK	Phase I/II clinical trial	Ariad
Neratinib/HKI-272	EGFR, HER2	Phase II clinical trial	Wyeth
AV412	EGFR, HER2	Phase I clinical trial	AVEO Pharmaceuticals
Lapatinib	EGFR, HER2	Phase III clinical trial	GSK

**Multiple signal transduction pathway inhibitors**			

XL647	EGFR, HER2, VEGFR	Phase II clinical trial	Exelixis
Vandetanib/caprelsa	EGFR, VEGFR2	Phase III clinical trial	AstraZeneca
BMS-690514	Pan-EGFR, VEGFR	Phase II clinical trial	Bristol-Myers Squibb

EGFR: epidermal growth factor receptor; NSCLC: non-small cell lung cancer; TKIs: tyrosine kinase inhibitors.

**Table 2:** Summary of ALK inhibitors for NSCLC.

Agent	Molecular properties	Approved status	Company
Xalkori/crizotinib	c-MET, ALK	Approved by FDA for patients with late-stage NSCLC carrying positive ALK.	Pfizer
AP26113	EGFR/T790M, ALK	Phase I/II clinical trial	Ariad
LDK378	ALK	Phase I clinical trial	Novartis
AF802/CH5424802	ALK	Phase I/II trial	Chugai
ASP3026	ALK	Phase I clinical trial	Astrella
X-396	ALK	Pre-clinic	Xcovery
GSK-1838705A	ALK	Pre-clinic	GSK
NMS-E628	ALK	Pre-clinic	-

ALK: anaplastic lymphoma kinase; EGFR: epidermal growth factor receptor; NSCLC: non-small cell lung cancer.

**Table 3:** The clinical trials in selected patients carrying EGFR mutation.

Author	Description	Drug	Patients (No. of patients)	End point	Results
**Compare EGFR tyrosine kinase inhibitors (TKIs) with chemotherapy in EGFR mutant patients**					

Rosell *et al*. [76]	Phase III (EURTAC)	Erlotinib *vs.* cisplatin/docetaxel (or gemcitabine)	EGFR^+^ (174)	PFS	PFS was 9.7 months (erlotinib) and 5.2 months (chemotherapy) (HR 0.37, *p* < 0.0001), the RR was 64% (erlotinib) and 18% (chemotherapy).
Zhou *et al*. [77**]**	Phase III (OPTIMAL)	Erlotinib *vs.* gemcitabine/carboplatin (GC)	EGFR^+^ (154)	PFS	PFS was 13.1 months (erlotinib) and 4.6 months (GC) (HR 0.16, *p* < 0.0001).
Mitsudomi *et al*. [78]	Phase III (WJTOG3405)	Gefitinib *vs.* cisplatin/docetaxel (CD)	EGFR^+^ (177)	PFS	PFS was 9.2 months (gefitinib) and 6.3 months (CD) (*p* < 0.0001).
Yang *et al*. [79]	Phase III (LUX-Lung 3)	Afatinib *vs.* pemetrexed/cisplatin	EGFR^+^ (345)	PFS	Prolonged PFS was found in afatinib group (11.1 *vs.* 6.9 months, HR 0.58, *p* = 0.0004).

Inoue *et al.* [80]	Phase III (NEJ002)	Gefitinib *vs. CBDCA+PTX (CP)*	EGFR^+^ (228)	OS	OS was not significant different between gefitinib and CP groups. The median survival time and 2-year survival rate were 27.7 months, 57.9% (gefitinib), and 26.6 months, 53.7% (CP) (HR 0.887; *p* = 0.483).

**EGFR TKIs was given alone in EGFR mutant patients: single arm design**					

Kim *et al*. [81]	Phase II	First-line gefitinib	EGFR^+^ (45)	Objective RR	Objective RR: 53.3%; DCR: 86.7%, the median PFS: 398 days; median OS: 819 days.

Tamura *et al*. [82]	Phase II (WJTOG0403)	First-line gefitinib	EGFR^+^ (28)	RR	The overall RR was 75%, the DCR was 96% and the median PFS was 11.5 months.

Sequist *et al*. [83]	Phase II	First-line gefitinib	EGFR^+^ (31)	RR	The RR was 55% and median PFS was 9.2 months.
Inoue *et al*. [84]	Phase II	First-line gefitinib	EGFR^+^ **(**22**)**	Overall RR	The overall RR was 66%, and the DCR was 90%, PS improvement rate was 79% (*p* < 0.00005), and 68% improved from ≥ PS 3 at baseline to ≤ PS 1. The median PFS, median survival time, and 1-year survival rate were 6.5 months, 17.8 months, and 63%.
Sugio *et al*. [85]	Phase II	Gefitinib monotherapy	EGFR^+^**(**19**)**	-	The overall RR, DCR, median PFS and median survival time were 63.2%, 89.5%, 7.1 months and 20 months.
Han *et al*. [86]	Phase II (ESTERN)	Erlotinib as neoadjuvant treatment	EGFR^+^ (5)	Radical resection rate	One male patient with stable disease after neoadjuvant treatment got right upper lobe resection.
Yang *et al*. [87]	Phase II (LUX-Lung 2)	First- or second-line afatinib	EGFR^+^ (129)	Objective RR	The objective RR was 66%.
Kris *et al*. [88]	Phase II	First-line dacomitinib (PF-00299804)	EGFR^+^ (47) or patients with adenocarcinoma, no prior systemic tx, had smoked <10 pack years.	PFS; PR	In the patients with mutant EGFR, PR rate was 74%. Preliminary PFS was 96% (4 months) and 77% (1 year). Preliminary median PFS was 17 months.

**Biomarker-based treatment selection: EGFR TKIs in the patients with positive mutation; chemotherapy in wild type patients**					

Zhong *et al*. [89]	Phase II (LUX-Lung 2)	EGFR^+^ in one arm given erlotinib, EGFR^−^ in another arm given GC	EGFR^+^ (24)	RR	The RR were 58% for the erlotinib arm and 33% for the GC arm (*p* = 0.49), the RRs were 17% for the erlotinib arm and 25% for the GC arm (*p* = 0.64).
Rosell *et al*. [90]	Phase II	EGFR^+^ were given erlotinib, and those with wild type EGFR received chemotherapy with or without cisplatin	EGFR^+^ (123)	-	Median survival exceeded 28 months for 12 patients with EGFR mutations, and 9–11 months for the patients with wild type EGFR. Two-year survival was 73.3% and 0%–41.2%, respectively.

**Second-generation EGFR TKIs in patients with resistance**					

Pietanza *et al*. [91]	Phase II	XL647	41 patients with relapsed or recurrent advanced NSCLC who progressed after ≥ 12 weeks of stable disease or response to erlotinib or gefitinib and/or those patients with a documented EGFR T790M	Objective RR	The objective RR was 3%, 67% of the patients harbored T790M had progression of disease, while14% of those without this mutation, 11 patients (28%) had a dose reduction due to toxicity.
Sequist *et al*. [92]	Phase II	Neratinib	167 patients with ≥ 12 weeks of prior TKI therapy of EGFR TKIs	Objective RR	The objective RR was 3% in EGFR mutant patients, 0% in the other patients.

DCR: disease control rate; EGFR: epidermal growth factor receptor; OS: overall survival; PFS: progression free survival; PS: performance score; PR: partial response; RR: response rate.

**Table 4:** The clinical trials in selected patients carrying wild type EGFR.

Author	Prescription	Drug and study design	Selection of patients	End point	Results
Kobayashi *et al*. [95]	Phase II	Erlotinib monotherapy	EGFR^−^ (31)	DCR and PFS	The RR, DCR, median PFS, and survival times were 17.2%, 44.8%, 2.1 months and 7.7 months, respectively.
Matsuura *et al*. [96]	Phase II	Erlotinib monotherapy	EGFR^−^ (20)	-	Overall RR was 15% and a DCR was 55%, median PFS and OS were 2.1 and 6.7 months, respectively.
Yoshioka *et al*. [97]	Phase II	Erlotinib monotherapy	EGFR^−^ (30)	Object RR	Object RR was 3.3%, and the disease became stable in 18 patients (60%), the median survival time and median PFS were 9.2 and 2.1 months, respectively.
Garassino *et al*. [98]	Phase III (TAILOR	Erlotinib *vs.* docetaxel	EGFR^−^ (211)	PFS	PFS was significant higher in docetaxel therapy (HR 0.70, *p* = 0.016) over erlotinib regimen.
Metro *et al*. [99]	-	Erlotinib or gefitinib monotherapy	EGFR^−^ (67)	PFS; OS	Median PFS and OS were 2.9 months and 18.0 months, respectively. KRAS mutant patients had significantly shorter PFS (1.6 months) than KRAS wild type patients (3.0 months) (*p* = 0.04).

DCR: disease control rate; EGFR: epidermal growth factor receptor; HR: hazard ratio; OS: overall survival; PFS: progression free survival; PR: partial response; RR: response rate.
